# Development of a TaqMan qPCR assay for trypanosomatid multi-species detection and quantification in insects

**DOI:** 10.1186/s13071-023-05687-3

**Published:** 2023-02-14

**Authors:** Olga Barranco-Gómez, Jessica Carreira De Paula, Jennifer Solano Parada, Tamara Gómez-Moracho, Ana Vic Marfil, María Zafra, Francisco José Orantes Bermejo, Antonio Osuna, Luis Miguel De Pablos

**Affiliations:** 1 Departamento de Parasitología, Grupo de Bioquímica y Parasitología Molecular CTS-183, Universidad de Granada, Granada, Spain; 2 Institute of Biotechnology, University of Granada, Granada, Spain; 3 Laboratorios Apinevada SL, Granada, Spain

**Keywords:** *Lotmaria*, *Crithidia*, *Leishmania*, Prevalence, Epidemiology, Honeybee, Diagnostic

## Abstract

**Background:**

Trypanosomatid parasites are widely distributed in nature and can have a monoxenous or dixenous life-cycle. These parasites thrive in a wide number of insect orders, some of which have an important economic and environmental value, such as bees. The objective of this study was to develop a robust and sensitive real-time quantitative PCR (qPCR) assay for detecting trypanosomatid parasites in any type of parasitized insect sample.

**Methods:**

A TaqMan qPCR assay based on a trypanosomatid-conserved region of the α-tubulin gene was standardized and evaluated. The limits of detection, sensitivity and versatility of the α-tubulin TaqMan assay were tested and validated using field samples of honeybee workers, wild bees, bumblebees and grasshoppers, as well as in the human infective trypanosomatid *Leishmania major*.

**Results:**

The assay showed a detection limit of 1 parasite equivalent/µl and successfully detected trypanosomatids in 10 different hosts belonging to the insect orders Hymenoptera and Orthoptera. The methodology was also tested using honeybee samples from four apiaries (*n* = 224 worker honeybees) located in the Alpujarra region (Granada, Spain). Trypanosomatids were detected in 2.7% of the honeybees, with an intra-colony prevalence of 0% to 13%. Parasite loads in the four different classes of insects ranged from 40.6 up to 1.1 × 10^8^ cell equivalents per host.

**Conclusions:**

These results show that the α-tubulin TaqMan qPCR assay described here is a versatile diagnostic tool for the accurate detection and quantification of trypanosomatids in a wide range of environmental settings.

**Graphical Abstract:**

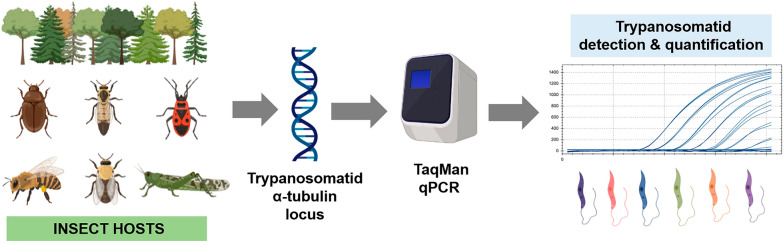

**Supplementary Information:**

The online version contains supplementary material available at 10.1186/s13071-023-05687-3.

## Background

Trypanosomatids (Euglenozoa: Kinetoplastea: Trypanosomatidae) are a widely distributed family of protozoan parasites that can infect numerous orders of insects [1, 2]. Parasites of the family Trypanosomatidae are classified on a nontaxonomic basis into two groups based on the number of hosts needed to complete development, with dixenous trypanosomatids (5 genera) having a life-cycle that requires infection of both an insect vector and a mammalian host and monoxenous trypanosomatids (19 genera) requiring only a singular, particular host, mainly insects in the orders Diptera, Hemiptera and Hymenoptera [1]. Monoxenous trypanosomatids have developed numerous strategies for transmission, such as cannibalism, predation, necrophagy, injection with saliva and fecal, oral or transovarial route [1, 3–5], and they can be found in adult-stage insects but also in larvae, pupae and or insect eggs. These parasites show diverse locations for thriving and proliferating within their insect hosts, such as the digestive tract, particularly the gut (foregut, midgut or hindgut), but also the Malpighian tubes and the hemolymph [3, 6, 7]. The wide diversity of typing units (TUs) found in trypanosomatids (> 300 so far) makes them one of the most successful groups of parasites in nature [4]. 

In this regard, several studies have demonstrated the continuous presence and dissemination of trypanosomatid parasites in insects. For example, a study on the presence of trypanosomatids in Austrian mosquitoes revealed that 12 out of 19 species harbored these parasites with a prevalence ranging from 16% to 61% [8]. In other studies, trypanosomatids were identified in eight species of *Drosophila* analyzed in southwest Ohio (USA), with a prevalence of 1–17% [9], and the prevalence of trypanosomatids on insects in the island of Curaçao and Papua New Guinea was 38% and 15%, respectively [10, 11]. In addition, the presence of monoxenous parasites has been identified in 4.3% of tsetse flies collected in the Dzanga-Sangha Protected Area (Central African Republic) [12]. Within this broad diversity of hosts, hymenopterans—and particularly bees—are a group of insects in which the presence of trypanosomatid parasites has been widely described. Bees are major pollinators involved in the maintenance of all terrestrial ecosystems, and they are currently experiencing a massive decline in numbers that is related to a wide variety of stressors, including habitat loss, climate change, pesticides and parasites [13].

Four species of trypanosomatid parasites have been identified to date (*Lotmaria passim, Crithidia mellificae, C. bombi* and *C. acanthocephali*) in bumblebees, wasps or honeybees [14–16]. These four parasite species have been found in hymenopteran samples across four continents, indicating the wide distribution of trypanosomatid flagellates across highly different ecological settings [17–23]. Trypanosomatid parasites have been associated with winter colony losses [21], reduced bumblebee colony fitness [24, 25] and/or reduced honeybee lifespan under experimental conditions [26], suggesting that they are a significant health threat to these insects.

To obtain a precise picture of the circulating levels of these parasites in nature, novel targets and molecular amplification assays are needed. The use of TaqMan probes versus SYBR green tracking dyes offers several advantages, such as higher specificity and sensitivity, which increases the chances for target detection. This detection sensitivity could be further increased by the use of multicopy gene families or of repeated DNA fragments as primers and probe targets [27, 28]. Sensitive and polyvalent multispecies detection assays are achieved using conserved regions that could capture the diversity of a particular group of organisms. The spliced leader RNA (SL-RNA), which consists of a conserved sequence of 39 bp added to the 5′ end of trypanosomatid pre-messenger RNA (mRNA) molecules, and the 28S ribosomal RNA (rRNA) locus have been used as targets for TaqMan real-time quantitative PCR (qPCR) assays aimed at detecting trypanosomatids in different blood samples, with limits of detection of 100 parasites per milliliter of blood [29, 30]. An alternative to these regions is the alpha/beta (α/β) tubulin array, a conserved multicopy trypanosomatid locus essential for the formation of the microtubular cytoskeleton. This α/β heterodimer is arranged in tandem repeats of gene copies varying in number between nine copies found in the *Leishmania mexicana* genome [31], 10 copies in *Leishmania tarentolae* [32], 12 copies in *Leishmania amazonensis* or *Leptomonas pyrrhocoris* [33, 34] and up to 19 copies in *Trypanosoma brucei* [35]. Therefore, this locus could be a good target for universal detection and quantification of trypanosomatid parasites. To date, honeybee trypanosomatid infections have been quantified using SYBR-green qPCR assays using as targets the small subunit rRNA (SSU rRNA) locus [6], the cytochrome* b* gene [36] or the internal transcribed spacer 1 (ITS1) [37]. However, no TaqMan qPCR assay has been developed yet for detection of trypanosomatid infections in insects. Therefore, the development of such an assay for diagnosis in bees or other insect hosts could be a powerful analytical tool.

Here we report our development of a novel TaqMan qPCR assay based on the amplification of the conserved α-tubulin gene fragment. The limits of detection, sensitivity and versatility of the α-tubulin TaqMan assay were tested and validated using field samples from honeybee workers, wild bees, bumblebees and grasshoppers, and the usefulness of the assay was demonstrated.

## Methods

### *Lotmaria passim* culture and field sampling

The trypanosomatid *L. passim* C1 strain was cultured in modified BHT media as previously described [38]. Genomic DNA (gDNA) was isolated from these cultures during the mid-log phase of growth (5 × 10^6^ cells/ml) using a standard phenol/chloroform/isoamyl protocol (see section Genomic DNA) and then used for generating qPCR standard curves.

During the field sampling, a total of 224 honeybees (approx. 28 bees/hive) from four apiaries (coded as 1–4) located in the Alpujarra region (south of Granada, Spain) were sampled in September 2018. Two hives (coded as A and B) were sampled in each apiary (Additional file 1: Table S1, Figures S1, S2). An additional 50 worker honeybees were collected from rosemary (*Rosmarinus officinalis*) flowers in the city center of Granada (37°08′48.3″N 3°36′28.2″W). Finally, 43 samples of wild bees, bumblebees and grasshoppers were collected during autumn 2021 and spring 2022 at different locations of Granada and Jaen provinces (Andalucía, Spain) as indicated in Additional file 1: Table S2. All insect samples were immediately frozen at − 20 °C before DNA extraction.

### Genomic DNA

Genomic DNA was extracted by crushing the abdomens of adult bees and larvae and incubating the samples overnight in lysis buffer and proteinase K at 55 °C. The gDNA was then purified using a standard phenol/chloroform/isoamyl protocol and treated with RNase A. The concentration and purity of the extracted DNA were determined on a Nanodrop 2000 spectrometer (Thermo Fisher Scientific, Waltham, MA, USA).

### α-Tubulin TaqMan qPCR

The primers and probes used for the TaqMan assay were designed to amplify a conserved region of the α-tubulin gene of trypanosomatid parasites (Additional file 1: Table S3, Figure S1A). Representative sequences of the α-tubulin gene from 23 trypanosomatid species were obtained from the TritrypDB database and aligned using ClustalW [39]. ePrimer3 software was used to design primers in the conserved 3′ end of the α-tubulin open reading frame (amplicon length: 193 bp), and the PrimerQuest™ Tool (Integrated DNA Technologies, Inc., Coralville, IA, USA) was used to design the TaqMan probe (Additional file 1: Table S3). BLAST analysis of the α-tubulin locus against organisms of the order Insecta resulted in no potential cross-reactions.

To check the versatility of the α-tubulin locus to amplify trypanosomatids, we performed a conventional PCR using the primers 198F/199R. Each reaction mixture contained 20 mM Tris-HCl, 3 mM MgCl2, 1 mM dNTPs, 0.2 µM of each primer (198F/119R), 3% kb Extender and Platinum® and 2 U of Taq DNA Polymerase (Thermo Fisher Scientific). The PCR amplification protocol consisted of an initial denaturation at 95° for 4 min, followed by 39 cycles at 95 °C for 30 s, 60 °C for 30 s and 72 °C for 20 s, with a final extension at 72 °C for 5 min. gDNA from *L. passim* cultures was used as the positive control, and autoclaved Milli-Q water was used as the negative control.

The qPCR reactions were performed using the TaqMan™ Fast Universal PCR Master Mix (1×), no AmpErase™ UNG (Applied Biosystems, Thermo Fisher Scientific) in a total reaction volume of 10 μl containing 900 nM of primers (198F-199R), 250 nM of α-tubulin probe and an average of 500 ng of DNA sample. The cycling conditions consisted of 95 °C for 5 min, followed by 40 cycles of 95 °C for 10 s and 60 °C for 25 s, in a CFX96 Real-Time System Thermocycler (Bio-Rad Laboratories, Hercules, CA, USA). The 18S gene was used as the qPCR internal control, using the oligonucleotides and conditions previously described, and reactions were performed in triplicate per sample.

### Quantification of trypanosomatid loads

Genomic DNA from “pseudo-infected” bees was used to construct the standard curves for qPCR quantification. This artificial sample was generated mixing non-infected bee gDNA with tenfold decreasing concentrations of *L. passim* gDNA. The corresponding copy number of α-tubulin/µl in the starting concentration was calculated using the following equation [40].

$$\alpha - {\text{tubulin }}\left( {{\text{copies}}/\mu {\text{L}}} \right) \, = {\text{ n}}_{{\alpha - {\text{tubulin}}}} * \, \left[ {\left( {{\text{C}}_{{{\text{gDNA}}}} *{\text{ Const}}_{{\text{A}}} } \right)/\left( {{\text{L}}_{{{\text{gDNA}}}} *{\text{ M}}_{{{\text{pb}}}} } \right)} \right]$$

where n_α-tubulin_ is the number of copies of α-tubulin in the diploid genome of *L. passim* (copies); C_gDNA_ is the concentration of *L. passim* gDNA (ng/µl); Const_A_ is the Avogadro constant (6.022 × 10^23^ pb/mol); L_gDNA_ is the size of the diploid genome of *L. passim* SF (PRA-403) strain (64 Mb [41]; and M_pb_ is the weight of a double-stranded base pair (average: 6.6 × 10^11^ ng/mol).

The standard curve was generated by tenfold dilutions of 2.24 × 10^7^ down to 2.24 copies/µl of α-tubulin of “pseudo-infected” bee gDNA. Each point of the standard curve was obtained from the mean value of three replicates and was considered acceptable only when all the replicates were positive. The cycle threshold (Ct) values of positive samples were obtained and copies were extrapolated from standard curves in each qPCR. To obtain the number of cell equivalents per sample, the copies/µl obtained in the unknown samples were divided by 24, which is the median number of copies of the α-tubulin gene in the diploid genome of the closest *Lotmaria passim* related trypanosomatid *Leptomonas pyrrhocoris* [34], and finally multiplied by the total sample volume in which the sample was resuspended. The analytical sensitivity was taken from the last dilution of the standard curve in which the triplicated parasite DNA was successfully measured.

### Sequencing

To determine the trypanosomatid species in the positive samples, the SSU rRNA gene fragment was amplified using the primers S762 and S763 according to Maslov et al. [42]. The sequences obtained have been deposited in the Genbank database under the accession numbers OP805899-OP805907.

## Results

### The trypanosome α-tubulin region is a pan-specific molecular target in different insect hosts

**Fig. 1:**
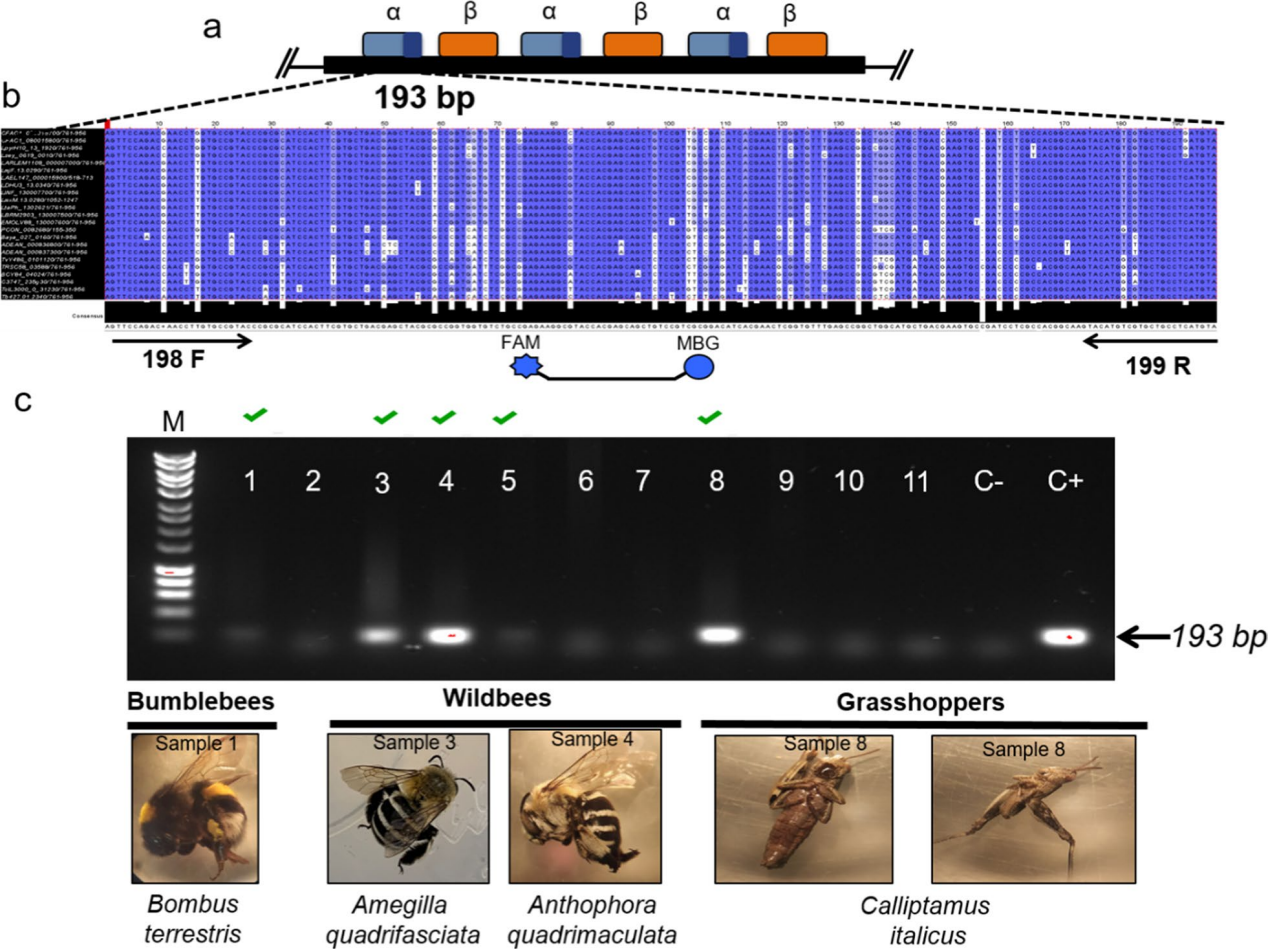
Trypanosomatid α-tubulin amplification in multiple insect hosts. **a** Illustration of the locus of trypanosomatid alpha/beta (α/β) tubulin tandem repeated arrays. As highlighted in dark blue, the 193-bp amplicon is located at the 3´end of each α-tubulin repeat, being the number of repeats variable across trypanosomatid species. **b** Clustal analysis of the 3′ end of the α-tubulin open reading frame showing conserved blocks of homology (in blue) that were used for designing primers and TaqMan probes. **c** Representative 2% agarose gel electrophoresis of the 193 α-tubulin trypanosomatid region amplified in samples of bumblebees, wild bees and grasshoppers. Numbers refer to different samples. The negative control (C-) and the positive control (C+) show negative and positive samples, respectively. F, Forward; FAM, dye label; M, molecular size marker; MGB, minor groove binder; R, reverse

The primers and probes were designed to target a conserved fragment of 193 bp of the α-tubulin gene, as shown in the Clustal analysis of 23 homolog genes in trypanosomatids (Fig. 1a, b). This region was tested and successfully amplified by conventional PCR giving a single amplicon using gDNA from *L. passim* cultures (data not shown).

To assay the performance of the 193-bp α-tubulin region as target for the PCR in various host species, a total of 43 samples comprising 27 wild bee, nine bumblebee and seven grasshopper species were tested for the presence of trypanosomatid parasites (Additional file 1: Table S2; Fig. 1c). The α-tubulin fragment was amplified in 21 out of 43 samples (48.8% of the examined samples), being found in 11 out of the 20 insect species analyzed (Additional file 1: Table S2). The positivity of the PCR was 42.8% (12/28) in solitary bees, 87.5% (7/8) in bumblebees and 28% (2/7) in grasshoppers (Additional file 1: Table S2). Of the positive samples, *C. bombi* (percentage of identity = 100%) from five different *Bombus terrestris* samples and *Lotmaria passim* (percentage of identity = 99.8%) from a bee of genus *Halictus* were successfully amplified and sequenced using a fragment of the SSU rRNA [42]. These data support the use of the 193-bp α-tubulin gene locus as a pan-specific molecular target for trypanosomatid identification.

### Analytical sensitivity of the α-tubulin TaqMan assay

Once the capacity of the α-tubulin TaqMan assay to amplify the 193-bp α-tubulin region in multiple insect hosts was verified, we used this region as a molecular target in “pseudo-infected” bee gDNA spiked with decreased concentrations of *Lotmaria passim* or *Leishmania major* DNA (1:10 dilution), to ascertain the efficiency and sensitivity of the TaqMan qPCR assay in different trypanosomatid species. The efficiency of the standard curve using *L. passim* gDNA was 99.7%, with* R*^2^ = 0.999 (*y* = − 3.328*x* + 39.395) and the last detected Ct of 35.72 ± 0.1 (Additional file 1: Figure S3A). The analytical sensitivity of the assay resulted in a limit of detection (LOD) of 24 α-tubulin copies/µl (= 1 cell equivalent/µl). The efficiency of the standard curve using *L. major* gDNA was 94.2%, with* R*^2^ = 0.996 (*y* = − 3.469*x* + 42.472) and the last detected Ct of 33.8 ± 0.1 (Additional file 1: Figure S3B). The analytical sensitivity of the assay resulted in a LOD of 240 α-tubulin copies/µl (= 10 cell equivalent/µL).

### Trypanosomatid infection levels in field-collected insect samples.

**Fig. 2:**
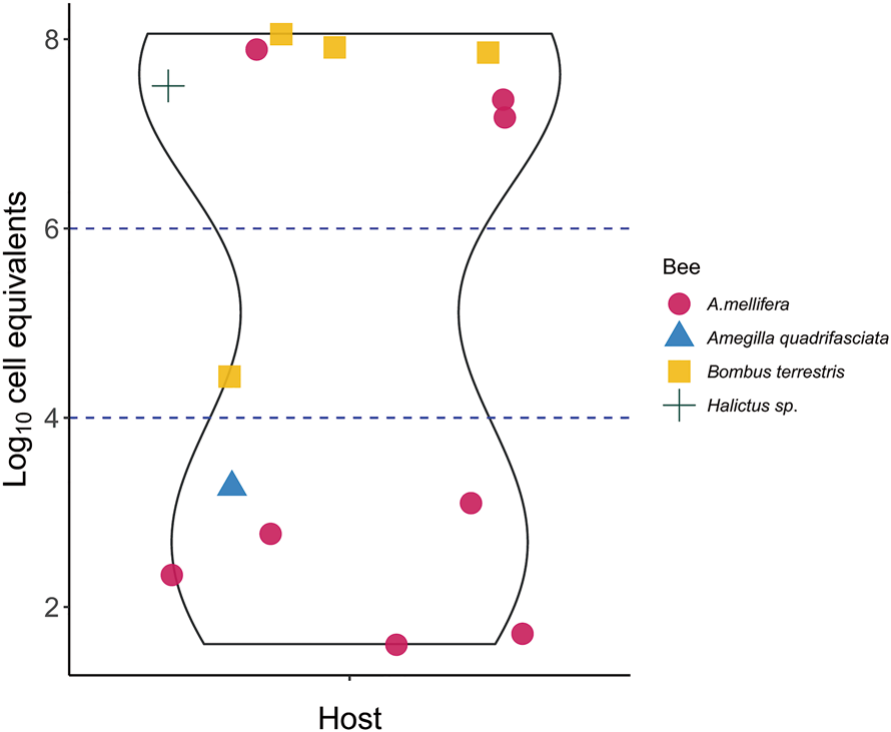
Trypanosomatid yields in the insect samples analyzed with the α-tubulin TaqMan assay. Data are presented as a violin plot showing the density of cell equivalents per insect. The red circles represent 8 infected samples of the western honey bee *Apis mellifera*, the yellow squares represent 4 infected samples of the buff-tailed bumblebee *Bombus terrestris*, the blue cross represents an infected sample of *Halictus* spp. and the blue triangle represents an infected white-banded digger bee *Amegilla quadrifasciata*. The blue dotted line represents 1 × 10^4^ cell equivalents per insect [Log4(10)] and 1 × 10^6^ cell equivalents per insect [Log6(10)]

To determine the analytical power of the TaqMan assay in different insect samples, we determined trypanosomatid infection levels (cell equivalents/bee) in honeybees and other pollinators. Honeybee samples (*n* = 224 samples) from four apiaries located in the south of Granada Province and in flowering plants in Granada city were collected, and trypanosomatid loads were quantified by qPCR. Trypanosomatids were detected in 2.68% (6/224) of the honeybee samples examined. Of the four apiaries analyzed, two apiaries (apiaries 1 and 2) were infected with trypanosomatids, with a positivity percentage ranging from 4.7% (1/21; colony 2A) to 13.3% (4/30; colony 1A). In comparison, trypanosomatid prevalence in honeybees feeding on flowering plants in Granada city was 4% (2/50). The quantification of the parasitemia varied from 40.6 to 7.7 × 10^7^ trypanosomatids per honeybee, which shows the disparity in infection levels found in nature (Fig. 2). The two positive bees analyzed from flowering plants in Granada city showed a high parasite loads of 2.3 × 10^7^ and 1.5 × 10^7^ parasites per bee, respectively (Fig. 2). The amplification and sequencing of the SSU rRNA [42] was only possible in three of the honeybee samples analyzed that tested positive for trypanosomatids, revealing the uniform presence of *Lotmaria passim* with a percentage of identity of 99.57% and 99.71% respectively.

To ascertain the capacity to measure trypanosomatid yields in other insect species, a selected group of insect hosts was chosen for this study. The quantification resulted in successful quantifications with variable yields ranging from 1.8 × 10^3^ parasites per sample for *Amegilla quadrifasciata* to the highest parasite loads in *Bombus terrestris* of 7.1 × 10^7^ parasites per insect (Fig. 2).

## Discussion

Trypanosomatid parasites are one of the most extended groups of parasites, with at least 24 genera, showing a high plasticity for thriving in insect hosts in many different ecological scenarios [1]. Determination of the prevalence of trypanosomatids in nature requires sensitive molecular tools for evaluating the ecology and distribution of this group of parasites. In the present study, we found trypanosomatid infection in 11 out of the 20 insect species examined based on amplification of the α-tubulin loci in a standard PCR, with an overall positivity of 48.8%. These results demonstrate the potential of this qPCR assay for analyzing trypanosomatid biodiversity in insect hosts. Among the insects found competent to harbor trypanosomatids, we found both solitary bees (genus *Anthophora*, *Amegilla* spp. and *Lasioglossum immunitum*) and social bees (*Halictus* spp., *Apis mellifera* and *B. terrestris*) and also non-hymenopteran insects such as grasshoppers (C*alliptamus italicus*)*.* Although there is a wide variety in the functional traits of the insects studied, such as body size, phenology, nesting location, sociality and/or diet, which could influence pathogen prevalence [43], our data support earlier findings that trypanosomatid infection [38, 43–45] can occur in both social and solitary species. For example, the α-tubulin TaqMan assay described herein detected for the first time trypanosomatid infection in bees belonging to the genus *Amegilla*, namely in a wild solitary bee also known as the white-banded Digger Bee. This medium-sized solitary bee usually looks for sunny slopes in sandy areas in various habitats and feeds on plants of the Verbenaceae, Lamiaceae and Boraginaceae families. Bees belonging to genus *Amegilla* have a wide distribution and can be found throughout Spain [46]. We also detected trypanosomatid infection in the grasshopper *C. italicus*. To our knowledge, only one study has shown the capacity of *Leishmania hertigi* to infect individuals of the order Orthoptera (*Schistocerca gregaria*), thereby inducing agglutination reactions in the hemolymph and further demonstrating that trypanosomes are capable of inducing defense reactions in this host [47].

Multicopy or repetitive gene sequences are generally used to increase the sensitivity of PCR assays [27, 28]. In the present study, the α-tubulin TaqMan assay had a detection limit of 24 copies of α-tubulin/µl, which represents 1 parasite equivalent/µl. Molecular methods for detecting multiple trypanosomatid species in insect samples have been developed using conserved regions of small and large subunits of rRNA genes [42], ITS sequences flanking the 5.8S rRNA gene [48], the SL-region or the 28S–large subunit (LSU) of the rRNA [29, 49]. Trypanosome nuclear genomes also contain an array of alternating α- and β-tubulin genes that can carry up to 19 copies per haploid genome in *T. brucei* [35]. The region between the α- and β-tubulin locus has been successfully used to design nested PCR for evaluating trypanosome infections in cattle [50]. However, however, there has been no report of a TaqMan qPCR assay for trypanosomatid detection and quantification based on this region. Thus far, TaqMan assays have been successfully applied for the diagnosis and quantification of specific trypanosomatid species in clinical samples. For example, infections with *T. cruzi* have been determined in infected human blood samples using kinetoplast DNA (kDNA) and nuclear satellite DNA targets with detection limits of 0.23 and 0.70 parasite equivalents/ml, respectively [51]. *Leishmania* species have been detected with a sensitivity of three copies of parasite per reaction using the mitochondrial cytochrome* c* oxidase I (COI; 10–20 copies in each mitochondrion) or the amino acid permease 3 (AAP3) (2 copies in the parasite genome) genes [52]. To date, few attempts have been made to obtain multispecies identification using TaqMan qPCR. For example, in two studies, the 28S, 18S and 5S rRNA targets showed a limit of 1000 to 100 trypanosomes per milliliter of blood in a diverse set of trypanosomatids; however, the assay was only tested in animal blood samples and was not validated for the detection of monoxenous trypanosomatid parasites in insect samples [29, 30]. The application of the α-tubulin TaqMan assay described in this article represents a reliable sensitive assay which would be an effective alternative method in that it showed a good dynamic range of detection in *L. passim* and *L. major* down to between 1 and 10 cell equivalents/µl, thereby allowing for the detection of low levels of circulating monoxenous and dixenous parasites in nature.

The quantitative measurement of parasite loads has important implications not only for assessing host health but also for evaluating changing host behavioral patterns, such as differences in secondary sex and male choice in birds or differences in plastic transmission between hosts and insect vectors. The effects exerted by trypanosomatids once inside their hosts can be many and diverse, from asymptomatic to symptomatic effects, depending on the species, the host and other factors, such as the presence of co-infections with other pathogens or physicochemical changes in the environment of the host [4]. For example, *Trypanosoma brucei* triggers the innate immune response and modifies feeding behavior in tsetse flies [53, 54], *Leishmania* spp. facilitates morphological changes in the stomodeal valve of sand fly hosts [55] and *Blastocrithidia gerrides* generates male skating endurance in water striders [56]. Since bees are threatened by different parasitic pests[13], the quantitative measurement of trypanosomatid yields could be an indicator of colony health status. In the present study we found two separate groups of parasite loads of insect hosts with parasite numbers < 1 × 10^4^ parasites and > 1 × 10^6^ trypanosomatids per insect, with the yields particularly high in three of the four samples of bumblebees. These data show the variability of infection intensities in the hosts, possibly reflecting different transmission capacities. In addition, our study shows the presence of *L. passim* in *Apis mellifera* and *Halictus* spp. The capacity for sharing hosts has been postulated as a potential driver of parasite spillover or spillback between wild bees and managed bees and is an important parasite trait that could influence parasite disease emergence [57]. Although the precise symptoms and pathology of trypanosomatids in bees are still unclear, it has been reported that honey bees * Osmia cornuta* and *Apis mellifera* experimentally infected with 2.5 × 10^4^ cells/bee and 4 × 10^4^ cells/bee of* C. mellificae* and* L. passim* showed increased mortality rates compared to uninoculated controls [26, 45], with parasite loads shown to range from 2 × 10^4^ parasites/bee up to 1 × 10^6^ parasites/bee using SYBR green qPCR [58, 59]. These data indicate that it is essential to monitor trypanosomatids in order to know the possible risks to infected honeybee colonies and to implement possible control measures in parasitized apiaries.

In conclusion, the use of highly sensitive and quantitative methods for trypanosomatid detection are crucial for detecting, controlling and preventing the spreading of trypanosomes and could help in determining the competence of insect hosts for transmitting such parasites and/or the impact of biotic and/or abiotic factors in pathogen dynamics. Based on the results of the present study, the α-tubulin TaqMan qPCR assay could be a useful analytical tool to ascertain the infection status in apiaries and other natural and experimental settings, allowing for the detection of trypanosomatid parasites in numerous niches and hosts in nature.

## Supplementary Information

**Additional file 1: Figure S1.** Location of the samples. Sampling was performed in 4 locations of Alpujarra region, south of Granada (Andalusia region, Spain). The figures were designed using mapchart software (https://www.mapchart.net/index.html) and Google maps (https://www.google.es/maps/?hl=es). **Figure S2.** Experimental design and sampling. Honeybee samples were collected from 4 apiaries (coded as 1-4) at Capileira, Torvizcón and Las Barreras, Alpujarra region locations situated at the Alpujarra region (south of Granada, Spain). Two random hives in each apiary (coded as A and B) were sampled. **Figure S3.** The analytical performance of the α-tub TaqMan assay in different trypanosomatid species was measured using standard amplification curves and linear regression curves. The efficiency and limits of detection were obtained using 7 serial fold dilutions of bee gDNA spiked with 2.4 × 107 copies/ul of *L. passim* down to 2.4 copies/µl of *L. passim* (**A**) or *L. major* (**B**) α-tubulin. **Table S1.** Number of honeybees collected from each hive at the different apiaries in Granada. **Table S2.** Wildbees, bumblebees and grasshoppers analyzed for the presence of trypanosomatid parasites. **Table S3.** Primers and probes sequences for qPCR assay to detect trypanosomatid parasites and insect DNA as an internal control.

## Data Availability

All data analyzed during this study are included in this published article.
