# Correction: Development of a TaqMan qPCR assay for trypanosomatid multi-species detection and quantification in insects

**DOI:** 10.1186/s13071-023-05723-2

**Published:** 2023-03-16

**Authors:** Olga Barranco-Gómez, Jessica Carreira De Paula, Jennifer Solano Parada, Tamara Gómez-Moracho, Ana Vic Marfil, María Zafra, Francisco José Orantes Bermejo, Antonio Osuna, Luis Miguel De Pablos

**Affiliations:** 1 Departamento de Parasitología, Grupo de Bioquímica y Parasitología Molecular CTS-183, Universidad de Granada, Granada, Spain; 2 Institute of Biotechnology, University of Granada, Granada, Spain; 3 Laboratorios Apinevada SL, Granada, Spain

**Correction: Parasites & Vectors (2023) 16:69** **https://doi.org/10.1186/s13071-023-05687-3**

Following publication of the original article [1], it was brought to the authors’ attention that the funding statement was incomplete: "This work was supported by the Spanish Programme for Knowledge Generation and Scientific and Technological Strengthening of the R + D + I System: ´Generación del Conocimiento 2018 (PGC2018-098929-A-I00 and PID2021-126938OB-I00"—the previous, incomplete funding information—has been corrected to "This work was supported by the Spanish Programme for Knowledge Generation and Scientific and Technological Strengthening of the R + D + I System: Proyecto generación del Conocimiento 2018 (PGC2018-098,929-A-I00) and proyecto PID2021.126938OB.I00 financiado por MCIN/AEI/10.13039/501100011033 y por FEDER Una manera de hacer Europa."

The funding has since been corrected in the original article.

The authors apologize for any inconvenience caused.
