# Supplementary material for: Development of a TaqMan qPCR assay for trypanosomatid multi-species detection and quantification in insects
Source: Parasit Vectors. 2023 Feb 14;16:69. doi: 10.1186/s13071-023-05687-3 (PMC9930332; doi:10.1186/s13071-023-05687-3)
Supplement: Supplementary file 1 — Additional file 1: Figure S1. Location of the samples. Sampling was performed in 4 locations of Alpujarra region, south of Granada (Andalusia region, Spain). The figures were designed using mapchart software (https://www.mapchart.net/index.html) and Google maps (https://www.google.es/maps/?hl=es). Figure S2. Experimental design and sampling. Honeybee samples were collected from 4 apiaries (coded as 1-4) at Capileira, Torvizcón and Las Barreras, Alpujarra region locations situated at the Alpujarra region (south of Granada, Spain). Two random hives in each apiary (coded as A and B) were sampled. Figure S3. The analytical performance of the α-tub TaqMan assay in different trypanosomatid species was measured using standard amplification curves and linear regression curves. The efficiency and limits of detection were obtained using 7 serial fold dilutions of bee gDNA spiked with 2.4 × 10^7^ copies/ul of L. passim down to 2.4 copies/µl of L. passim (A) or L. major (B) α-tubulin. Table S1. Number of honeybees collected from each hive at the different apiaries in Granada. Table S2. Wildbees, bumblebees and grasshoppers analyzed for the presence of trypanosomatid parasites. Table S3. Primers and probes sequences for qPCR assay to detect trypanosomatid parasites and insect DNA as an internal control. [file 13071_2023_5687_MOESM1_ESM.doc]

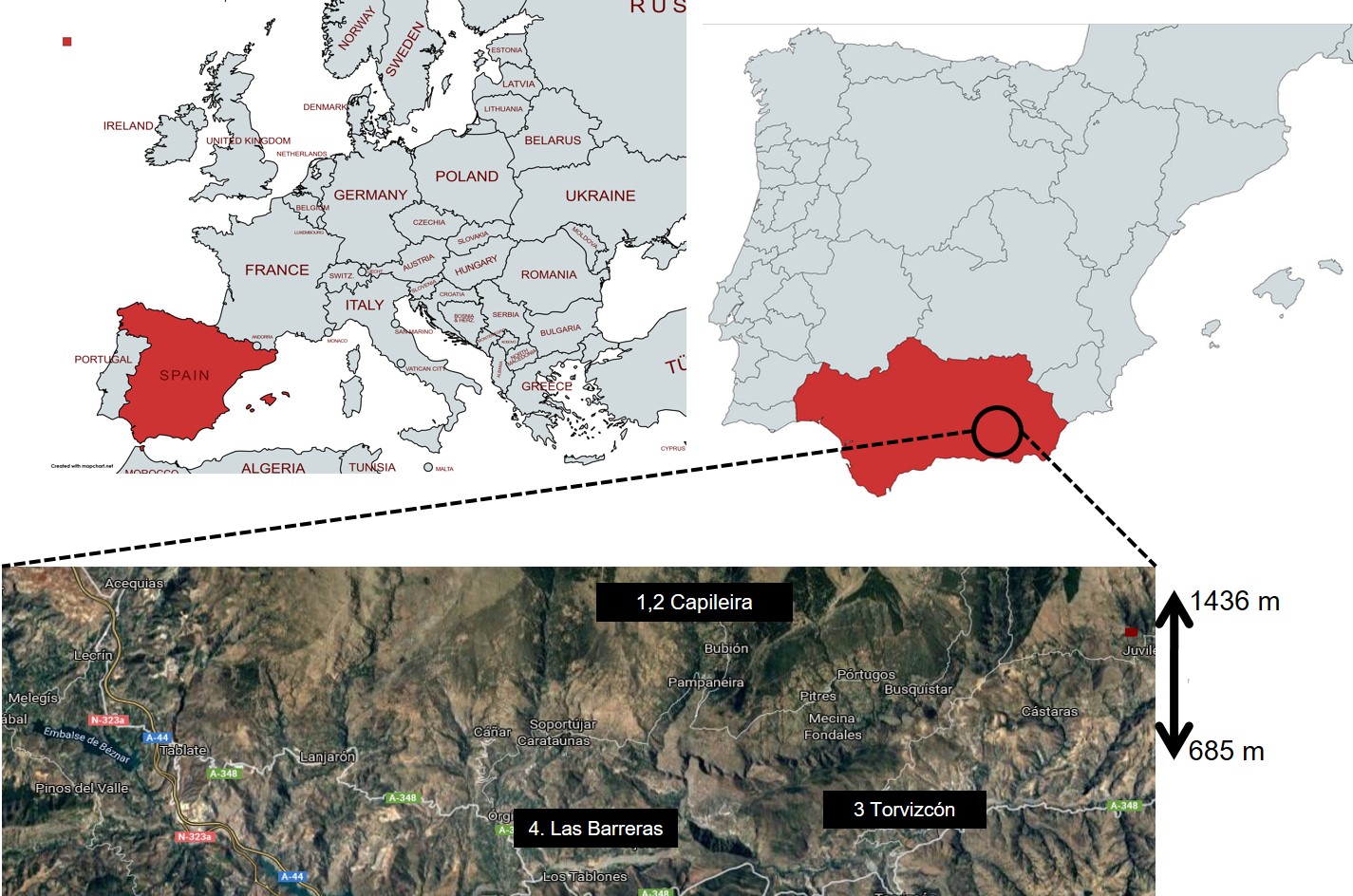


Additional file 1: Figure S1. Location of the samples. Sampling were performed in 4 locations of Alpujarra region at the south of Granada (Andalusia region, Spain). The figures were performed using mapchart software (<https://www.mapchart.net/index.html>) and google maps (<https://www.google.es/maps/?hl=es>).


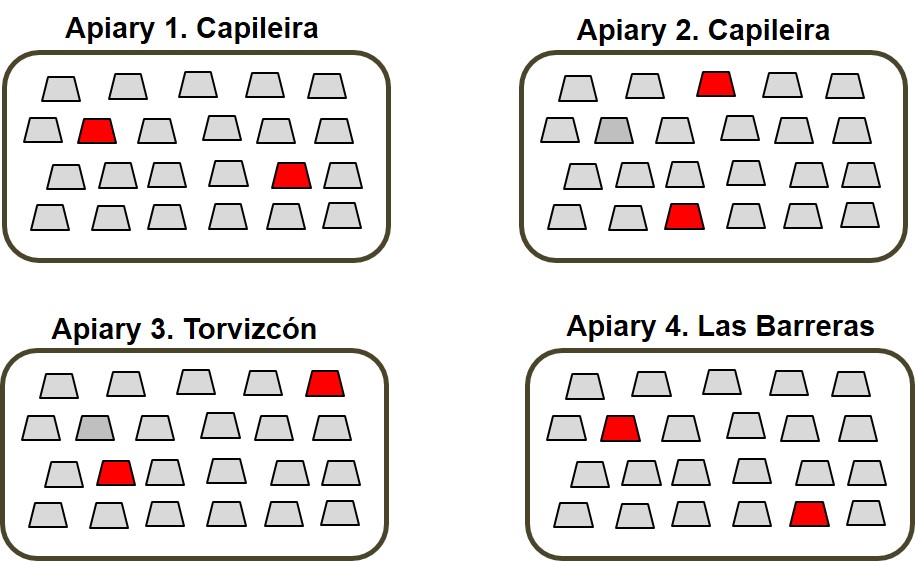


Additional file 1: Figure S2. Experimental design and sampling. Honeybee samples were collected from 4 apiaries (coded as 1-4) at Capileira, Torvizcón and Las Barreras. Alpujarra region locations situated at the Alpujarra region (South of Granada, Spain). Two random hives in each apiary (coded as A and B) were sampled.


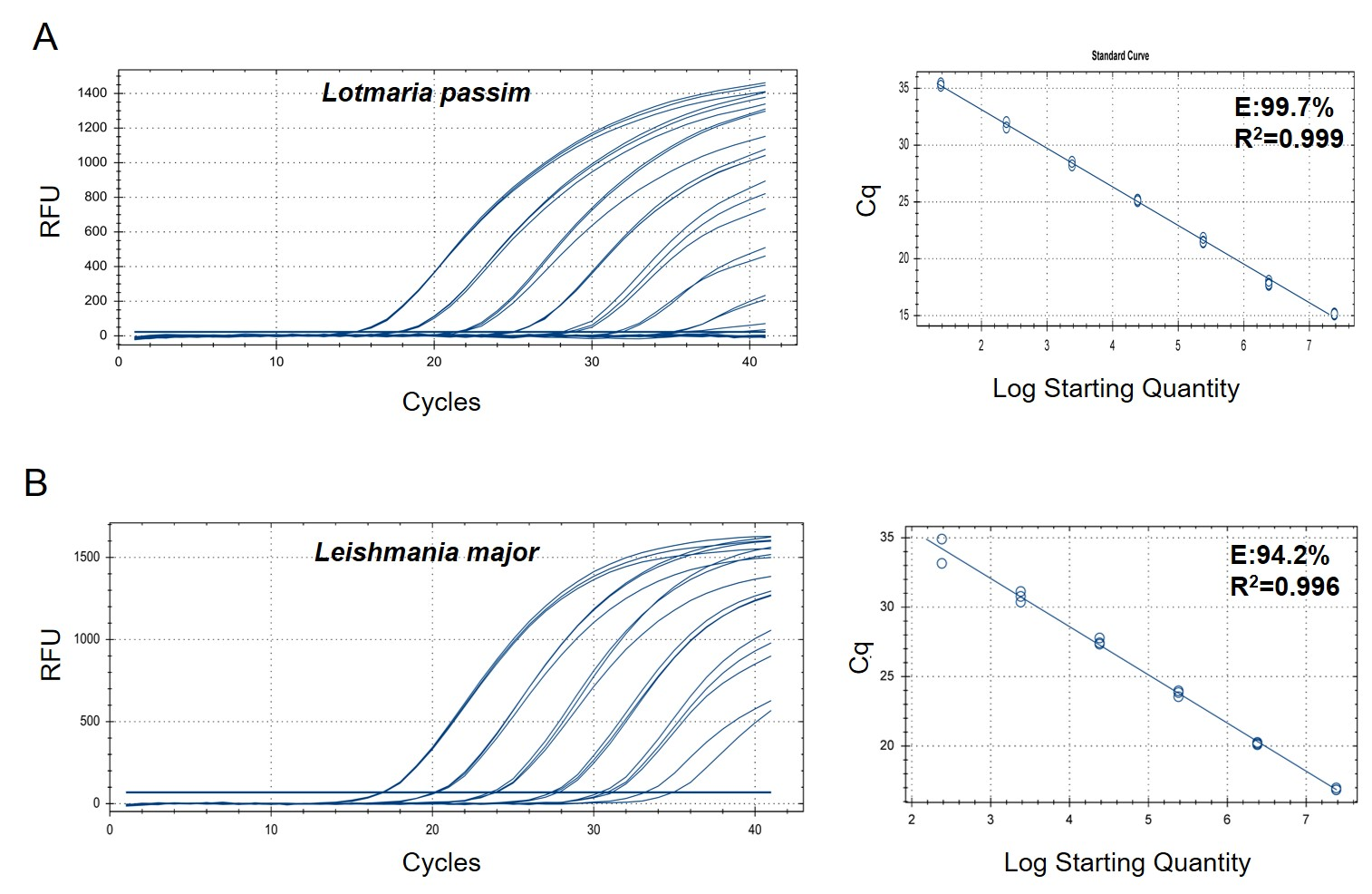


**Additional file 1: Figure S3**. The analytical performance of the α-tub TaqMan assay in different trypanosomatid species was measured using standard amplification curves and linear regression curves. The efficiency and limits of detection were obtained using 7 serial fold dilutions of bee gDNA spiked with 2.4 x 10^7^ down to 2.4 copies/µL of *L. passim* (A) or *L. major* (B) α-tubulin.

Additional file 1: Table S1. Number of honeybees collected from each hive at the different apiaries in Granada.

| Number of sample | Location | Hive A  (n) | Hive B  (n) |
| --- | --- | --- | --- |
| 1 | Barreras (36°55'25.1"N 3°31'42.9"W) | 30 | 30 |
| 2 | Torvizcón (36°52'45.0"N 3°17'46.5"W) | 21 | 23 |
| 3 | Capileira (36°57'40.4"N 3°21'35.5"W). | 30 | 30 |
| 4 | Capileira (36°57'40.4"N 3°21'35.5"W). | 30 | 30 |

Additional file 1: Table S2. Wildbees, bumblebees and grasshoppers analyzed for the presence of trypanosomatid parasites.

| Sample | Order | *Specie* | Location | Latitude | Longitude | Date | α-tub PCR +/- |
| --- | --- | --- | --- | --- | --- | --- | --- |
| 1 | Hymenoptera | *Anthophora quadrimaculata* | Armilla | 37°08'38.5"N+ | 3°36'58.4"W | 24/10/2021 | + |
| 2 | Hymenoptera | *Anthophora quadrimaculata* | Armilla | 37°08'38.9"N | 3°36'57.6"W | 24/10/2021 | - |
| 3 | Hymenoptera | *Anthophora plumipes* | Granada | 37°09'30.9"N | 3°36'39.9"W | 10/02/2022 | + |
| 4 | Hymenoptera | *Anthophora plumipes* | Granada | 37°09'25.3"N | 3°36'45.4"W | 10/02/2022 | - |
| 5 | Hymenoptera | *Antophora bimaculata* | Guadix | 37°05'18.36"N | 3°32´05.6098W | 29/11/2021 | + |
| 6 | Hymenoptera | *Amegilla quadrifasciata* | Granada | 37°05'18.36"N | 3°32´05.6098W | 28/10/2021 | + |
| 7 | Hymenoptera | *Amegilla quadrifasciata* | Granada | 37°05'18.36"N | 3°32´05.6098W | 28/10/2021 | + |
| 8 | Hymenoptera | *Amegilla quadrifasciata* | Granada | 37°05'18.36"N | 3°32´05.6098W | 28/10/2021 | + |
| 9 | Hymenoptera | *Amegilla quadrifasciata* | Granada | 37°05'18.36"N | 3°32´05.6098W | 28/10/2021 | + |
| 10 | Hymenoptera | *Bombus terrestris* | Granada | 37°10'07.7"N | 3°35'43.7"W | 02/02/2022 | + |
| 11 | Hymenoptera | *Bombus terrestris* | Granada | 37°10'15.4"N | 3°36'34.2"W | 03/02/2022 | + |
| 12 | Hymenoptera | *Bombus terrestris* | Granada | 37°10'36.84"N | 3°36´50.04W | 18/02/2022 | + |
| 13 | Hymenoptera | *Bombus terrestris* | Granada | 37°10'36.84"N | 3°36´50.04W | 18/02/2022 | + |
| 14 | Hymenoptera | *Bombus terrestris* | Granada | 37°10'36.84"N | 3°36´50.04W | 18/02/2022 | + |
| 15 | Hymenoptera | *Bombus terrestris* | Monachil | 37°05'37.23"N | 3°32´12.46W | 02/03/2022 | + |
| 16 | Hymenoptera | *Bombus terrestris* | Granada | 37°11'18.5"N | 3°36'12.7"W | 16/02/2022 | + |
| 17 | Hymenoptera | *Bombus terrestris* | La Carolina | 38°16'52.4"N | 3°36'40.2"W | 03/04/2022 | - |
| 18 | Hymenoptera | *Bombus terrestris* | La Carolina | 38°16'52.3"N | 3°36'40.0"W | 31/03/2022 | - |
| 19 | Hymenoptera | *Osmia cornuta* | Granada | 37°11'18.9"N | 3°36'12.9"W | 16/02/2022 | - |
| 20 | Hymenoptera | *Osmia cornuta* | Granada | 37°11'18.4"N | 3°36'12.8"W | 23/02/2022 | - |
| 21 | Hymenoptera | *Osmia rufa* | Granada | 37°11'28.1"N | 3°36'24.2"W | 02/03/2022 | - |
| 22 | Hymenoptera | *Osmia rufa* | Granada | 37°11'28.0"N | 3°36'24.1"W | 28/03/2022 | - |
| 23 | Hymenoptera | *Osmia leaiana* | La Carolina | 38°16'51.6"N | 3°36'42.1"W | 02/04/2022 | - |
| 24 | Hymenoptera | *Eucera longiscornis* | Monachil | 37°05'18.36"N | 3°32´05.6098W | 02/03/2022 | + |
| 25 | Hymenoptera | *Eucera nigrescens* | Granada | 37°10'37.3"N | 3°36'50.4"W | 17/02/2022 | - |
| 26 | Hymenoptera | *Eucera nigrescens* | Granada | 37°10'37.2"N | 3°36'50.0"W | 17/02/2022 | - |
| 27 | Hymenoptera | *Andrena nigroaenea* | Granada | 37°11'18.3"N | 3°36'12.8"W | 22/02/2022 | - |
| 28 | Hymenoptera | *Andrena denticulata* | La Carolina | 38°16'52.0"N | 3°36'41.7"W | 02/04/2022 | - |
| 29 | Hymenoptera | *Andrena flavipes* | Monachil | 37°05'37.23"N | 3°32´12.46W | 02/03/2022 | + |
| 30 | Hymenoptera | *Andrena barbilabris* | La Carolina | 38°16'52.2"N | 3°36'39.5"W | 03/04/2022 | - |
| 31 | Hymenoptera | *Megachile pilidens* | Monachil | 37°05'18.24"N | 3°32´05.60W | 22/10/2021 | - |
| 32 | Hymenoptera | *Halictus* spp*.* | Atarfe | 37°24´02.24"N | 3°54´91.35W | 01/03/2020 | + |
| 33 | Hymenoptera | *Lasioglossum immunitum* | Durcal | 37°00'19.40"N | 3°33´59.29W | 23/10/2021 | - |
| 34 | Hymenoptera | *Lasioglossum immunitum* | Granada | 37°10'53.04"N | 3°37´03.39W | 31/10/2021 | - |
| 35 | Hymenoptera | *Lasioglossum immunitum* | Granada | 37°10'36.84"N | 3°36´50.04W | 18/02/2022 | + |
| 36 | Hymenoptera | *Colletes abeillei* | Monachil | 37°05'18.36"N | 3°32´05.60W | 22/10/2021 | + |
| 37 | Orthoptera | *Calliptamus ithalicus* | La Zubia | 37°06'47.52"N | 3°34´39.80W | 03/04/2022 | + |
| 38 | Orthoptera | *Calliptamus ithalicus* | La Zubia | 37°06'47.52"N | 3°34´39.80W | 03/04/2022 | + |
| 39 | Orthoptera | *Calliptamus ithalicus* | La Zubia | 37°06'47.52"N | 3°34´39.80W | 03/04/2022 | - |
| 40 | Orthoptera | *Calliptamus ithalicus* | La Zubia | 37°06'47.52"N | 3°34´39.80W | 03/04/2022 | - |
| 41 | Orthoptera | *Chorthippus biguttulus* | La Zubia | 37°06'47.52"N | 3°34´39.80W | 03/04/2022 | - |
| 42 | Orthoptera | *Chorthippus biguttulus* | La Zubia | 37°06'47.52"N | 3°34´39.80W | 03/04/2022 | - |
| 43 | Orthoptera | *Chorthippus biguttulus* | La Zubia | 37°06'47.52"N | 3°34´39.80W | 03/04/2022 | - |

Additional file 1: Table S3. Primers and probes sequences for qPCR assay to detect trypanosomatid parasites and insect DNA as an internal control.

| **Target** | **Gene** | **Oligos** | **Sequence (5´- 3´)** | **Conc. (nM)** |
| --- | --- | --- | --- | --- |
| Trypanosomatids | α-tub | 198F | AGTTCCAGACGAACCTGGTG | 250 |
|  |  | 199R | TACATCAGGCAGCACGACAT | 250 |
|  |  | Tryp α -tub probe | FAM-AAGGCGTACCACGAGCAGCT-MGB | 900 |
| Hymenoptera [40] | 18S | 18SF | TAACTGGCATTATGTGGTACGTC | 100 |
|  |  | 18F | CCTCGACACTCAGTGAAGAGC | 100 |
|  |  | 18S Probe | Cy5-AGCTCCTYGCGGGCGGTCCAA- BHQ1 | 200 |
